# Supplementary material for: Trifolium repens-Associated Bacteria as a Potential Tool to Facilitate Phytostabilization of Zinc and Lead Polluted Waste Heaps
Source: Plants (Basel). 2020 Aug 6;9(8):1002. doi: 10.3390/plants9081002 (PMC7466184; doi:10.3390/plants9081002)
Supplement: Supplementary file 1 [file plants-09-01002-s001.pdf]

plants

MDPI

Supplementary Tables and Figures

Table 1. Variable sites in 900-bp long fragments of 16S rRNA gene of *Bacillus megaterium* genotypes from the Zn-Pb rich waste heaps in Bolesław, Saturn, and Bukowno and the non-metalliferous reference area in Bolestraszyce.

Genotype	Number of variable site						Frequency [*f*]	Strains
	9	16	40	47	72	511		
A	T	T	G	C	C	A	0.10	BolL EW1_G05, SatL EW2
B	C	.	C	.	.	G	0.15	BolL EW3_A02, BolL EW3_C03, SatL EW3
C	C	.	C	.	.	.	0.30	BolL EW3_D03, BukL EW3_H07, BukL EW3_C01, BolR EW3_A03, SatR EW1, SatR EW2
D	.	G	.	.	.	.	0.05	BceL EW1_D03
E	.	G	.	T	.	.	0.15	BceL EW1_G02, BceL EW1_G04, BukR EW1_A09
F	.	.	C	.	.	.	0.20	BceL EW1_C01, BukR EW2_H09, BukR EW1_D11, BceN EW2_F01
G	.	G	.	T	T	.	0.05	BukR EW1_G08

Table 2. Variable sites in 900-bp long fragments of 16S rRNA gene of *Stenotrophomonas maltophilia* genotypes from the Zn-Pb rich waste heaps in Bolesław, Saturn, and Bukowno and the non-metalliferous reference area in Bolestraszyce.

Genotype	Number of variable site																		Frequency [*f*]	Strains
	93	104	113	114	115	117	128	134	145	182	273	525	573	586	619	628	686	688		
A	C	G	T	T	G	G	T	C	T	C	A	T	G	A	G	T	G	G	0.154	BolL EW3_B05, SatL EW1
B	.	.	.	.	.	.	A	.	.	.	T	C	A	.	.	.	A	A	0.077	BolL EW3_C04,
C	.	.	.	.	.	.	.	.	.	.	.	G	.	T	.	.	G	G	0.077	BolN EW2_D05
D	.	.	.	.	.	.	.	.	.	.	.	.	.	.	A	.	A	.	0.154	BolN EW3_G01, SatN EW3
E	.	.	.	.	.	.	.	.	.	.	.	.	.	.	A	.	.	.	0.077	BolN EW3_B03
F	.	.	.	.	.	.	.	.	.	.	.	.	.	.	G	.	.	.	0.077	BukN EW3_F07
G	.	.	.	.	.	.	.	.	.	T	.	.	.	.	.	.	.	.	0.154	BolS EW2_E08, SatS EW1
H	.	C	.	G	A	A	.	T	.	T	.	.	.	.	.	.	.	.	0.077	BukS EW1_H09
I	.	.	.	G	.	.	.	.	.	C	.	.	.	.	.	.	.	.	0.077	BceS EW2_D08
J	.	.	.	.	.	.	.	.	.	T	.	.	.	.	.	.	.	.	0.077	BceS EW2_F10

Table 3. Potential plant-growth promoting traits of strains isolated from the endosphere tissues (*i.e.* roots, nodules, and leaves) and the rhizosphere soil of *T. repens* originating from the Bolesław, Saturn, and Bukowno waste heaps, and the non-metalliferous reference area in Bolestraszyce. Abbreviations: IAA — indole-3-acetic acid synthesis, sid — siderophores production, org acids — organic acids production, ACCD — deaminase ACC activity, acet — acetoin production, P solub — phosphorus solubilization, N-fix — atmospheric nitrogen fixation.

				Plant-growth promotion traits							Metal tolerance						
**No**	**Bacterial strain ID**	***T. repens* isol. source**	**NCBI accession number**	**IAA**	**sid**	**org acids**	**ACCD**	**acet**	**P solub**	**N-fix**	Cd 0.4 mM	Cd 0.8 mM	Zn 0.6 mM	Zn 1.0 mM	Cu 0.4 mM	Cu 0.6 mM	Pb 2.0 mM
1	*Bacillus subtilis* BolS EW2_A11	soil	MN943580	0	1	0	1	1	0	0	0	0	1	0	0	0	0
2	*Citrobacter agglomerans* BukS EW2_E01	soil	MN943589	1	1	0	1	1	1	1	1	1	1	1	1	1	1
3	*Citrobacter* sp. BukS EW2_D02	soil	MN943590	1	1	0	1	1	1	1	1	0	1	1	1	1	1
4	*Enterobacter* sp. BceS EW2_C06	soil	MN943599	1	1	0	1	1	1	0	1	1	1	1	1	1	1
5	*Enterobacter* sp. BceS EW2_D06	soil	MN943592	1	1	0	1	1	1	1	1	1	1	1	1	1	1
6	*Enterobacter* sp. BceS EW2_G08	soil	MN943593	1	1	0	1	1	1	1	1	1	1	1	1	1	1
7	*Enterobacter* sp. BolS EW3_C02	soil	MN943584	1	1	0	1	1	1	0	1	1	1	1	1	1	1
8	*Kosakonia cowanii* BceS EW2_C10	soil	MN943594	1	1	0	1	1	1	0	1	1	1	1	1	1	1
9	*Lelliottia* sp. BukS EW3_D02	soil	MN943591	1	1	0	1	1	1	1	0	0	1	1	1	1	1
10	*Pseudomonas protegens* BolS EW2_E10	soil	MN943582	1	1	0	1	1	1	0	1	1	1	1	1	1	1
11	*Pseudomonas protegens* BolS EW2_F08	soil	MN943583	1	1	0	1	1	1	0	0	0	0	0	0	0	0
12	*Pseudomonas putida* BukS EW1_G10	soil	MN943587	1	1	0	1	0	1	0	1	1	1	1	1	1	1
13	*Pseudomonas putida* BukS EW2_C03	soil	MN943588	1	1	1	1	1	1	1	1	1	1	1	1	1	1
14	*Pseudomonas* sp. BceS EW2_C08	soil	MN943597	1	1	0	0	1	1	1	1	1	1	1	1	1	1
15	*Pseudomonas* sp. BukS EW1_A10	soil	MN943586	1	1	0	1	0	1	0	0	0	0	0	0	0	0
16	*Serratia plymuthica* BceS EW2_H08	soil	MN943598	1	1	0	1	1	1	1	0	0	1	1	1	1	1
17	*Stenotrophomonas maltophilia* BukS EW1_H09	soil	MN943585	1	1	0	0	0	1	0	0	0	0	0	0	0	0
18	*Stenotrophomonas* sp. BceS EW2_D08	soil	MN943595	1	1	0	1	1	1	1	1	1	1	1	1	1	1
19	*Stenotrophomonas* sp. BceS EW2_F10	soil	MN943596	1	1	0	1	1	1	0	1	1	1	1	1	1	1
20	*Stenotrophomonas* sp. BolS EW2_E08	soil	MN943581	1	1	0	1	1	1	1	0	0	0	0	0	0	0
21	*Stenotrophomonas maltophilia* SatS EW1	soil	MN943610	1	1	0	1	1	1	1	0	0	0	0	0	0	0
22	*Pseudomonas protegens* SatS EW2	soil	MN943611	1	1	0	1	1	1	0	1	1	1	1	1	1	1

				Plant-growth promotion traits							Metal tolerance						
**No**	**Bacterial strain ID**	***T. repens* isol. source**	**NCBI accession number**	**IAA**	**sid**	**org acids**	**ACCD**	**acet**	**P solub**	**N-fix**	Cd 0.4 mM	Cd 0.8 mM	Zn 0.6 mM	Zn 1.0 mM	Cu 0.4 mM	Cu 0.6 mM	Pb 2.0 mM
23	*Lelliottia amnigena* SatS EW3	soil	MN943612	1	1	0	1	1	1	1	0	0	1	1	1	1	1
24	*Bacillus drentensis* BceR EW2_B05	root	MN943555	1	1	0	1	1	1	0	1	1	1	1	1	1	1
25	*Bacillus megaterium* BolR EW3_A03	root	MN943541	1	1	1	1	1	1	1	1	1	1	1	1	1	1
26	*Bacillus megaterium* BukR EW1_A09	root	MN943547	1	1	0	1	0	1	0	1	1	1	0	1	1	1
27	*Bacillus megaterium* BukR EW2_H09	root	MN943548	1	1	1	1	0	1	1	1	1	1	1	1	1	1
28	*Bacillus simplex* BceR EW2_A05	root	MN943554	1	1	0	1	1	1	0	1	1	1	1	1	1	1
29	*Bacillus simplex* BceR EW2_H05	root	MN943553	1	1	0	1	0	1	0	0	0	1	0	0	0	0
30	*Bacillus* sp. BolR EW2_E07	root	MN943542	1	1	1	1	1	1	1	1	1	1	1	1	1	1
31	*Bacillus* sp. BukR EW1_D11	root	MN943549	0	1	0	1	1	1	1	0	0	0	0	0	0	0
32	*Bacillus* sp. BukR EW1_E11	root	MN943550	1	1	0	1	1	1	0	0	0	0	0	0	0	0
33	*Bacillus* sp. BukR EW1_G08	root	MN943551	1	1	0	1	1	1	1	0	0	1	1	1	1	1
34	*Bacillus thuringiensis* BolR EW1_H10	root	MN943543	1	1	0	1	1	1	0	0	0	1	0	1	1	1
35	*Bacillus thuringiensis* BolR EW2_G01	root	MN943544	1	1	0	1	1	1	0	1	1	0	0	0	0	0
36	*Burkholderia* sp. BceR EW2_C01	root	MN943559	1	1	1	1	1	1	1	0	1	1	1	1	1	1
37	*Leucobacter* sp. BukR EW1_B12	root	MN943552	0	1	1	1	1	1	0	0	0	1	1	1	1	1
38	*Microbacterium foliorum* BceR EW2_C07	root	MN943557	1	1	1	1	1	1	0	1	1	1	1	1	1	1
39	*Microbacterium* sp. BolR EW2_A06	root	MN943545	1	1	0	1	1	1	0	0	1	0	0	0	0	0
40	*Microbacterium* sp. BolR EW2_B12	root	MN943546	1	1	0	1	1	1	0	0	0	0	0	0	0	0
41	*Pantoea agglomerans* BceR EW2_G11	root	MN943558	1	0	0	1	1	1	1	1	1	1	1	1	1	1
42	*Pseudomonas corrugata* BceR EW2_E11	root	MN943556	1	1	0	1	1	1	0	1	0	1	1	1	1	1
43	*Stenotrophomonas maltophilia* BukN EW3_F07	root	MN943573	1	1	0	1	1	1	0	1	1	1	1	1	1	1
44	*Bacillus megaterium* SatR EW1	root	MN943603	1	1	1	1	0	1	1	1	1	1	1	1	1	1
45	*Microbacterium* sp. SatR EW2	root	MN943604	1	1	0	1	1	1	0	1	1	0	0	0	0	0
46	*Bacillus megaterium* SatR EW3	root	MN943605	1	1	1	1	1	1	1	1	1	1	1	1	1	1
47	*Bacillus megaterium* BceN EW2_F01	nodule	MN943575	1	1	0	1	1	1	1	1	1	1	1	1	0	0
48	*Bacillus simplex* BceN EW2_F05	nodule	MN943577	1	1	0	1	1	1	1	1	1	1	1	1	1	1
49	*Bacillus* sp. BceN EW1_G07	nodule	MN943576	1	1	0	1	1	0	1	0	0	0	0	0	0	0

No	Bacterial strain ID	*T. repens* isol. source	NCBI accession number	Plant-growth promotion traits							Metal tolerance						
				IAA	sid	org acids	ACCD	acet	P solub	N-fix	Cd 0.4 mM	Cd 0.8 mM	Zn 0.6 mM	Zn 1.0 mM	Cu 0.4 mM	Cu 0.6 mM	Pb 2.0 mM
50	*Bacillus thuringiensis* BolN EW1_H12	nodule	MN943560	0	0	0	1	1	1	0	0	0	0	0	1	1	1
51	*Bacillus thuringiensis* BolN EW2_A04	nodule	MN943561	1	1	1	1	1	1	0	1	0	1	0	0	0	0
52	*Bacillus thuringiensis* BolN EW2_B01	nodule	MN943562	1	1	0	1	1	1	0	1	1	1	1	0	0	0
53	*Bacillus thuringiensis* BolN EW2_H03	nodule	MN943563	1	1	0	1	1	0	0	0	0	1	0	0	0	0
54	*Bacillus thuringiensis* BukN EW2_D01	nodule	MN943571	1	1	1	1	1	1	1	0	0	0	0	0	0	0
55	*Bacillus thuringiensis* BukN EW2_E12	nodule	MN943572	1	1	0	1	0	1	0	0	0	0	0	0	0	0
56	*Chryseobacterium* sp. BolN EW3_E01	nodule	MN943564	1	1	1	1	1	0	1	1	1	1	1	1	1	1
57	*Erwinia persicina* BceN EW3_A04	nodule	MN943579	1	1	1	1	1	1	1	1	0	1	1	0	0	0
58	*Flavobacterium* sp. BceL EW2_A12	nodule	MN943538	1	0	1	1	0	0	0	0	0	0	0	0	0	0
59	*Micrococcus luteus* BukN EW2_C04	nodule	MN943574	1	1	0	1	1	1	1	1	1	1	1	1	1	1
60	*Pseudomonas* sp. BceN EW3_D01	nodule	MN943578	1	1	1	1	1	1	1	1	1	1	1	1	1	1
61	*Pseudomonas* sp. BolN EW1_D09	nodule	MN943568	1	1	0	1	1	1	1	0	0	1	1	1	0	0
62	*Pseudomonas* sp. BolN EW1_G12	nodule	MN943570	1	1	0	0	0	1	0	1	1	1	1	1	1	1
63	*Pseudomonas* sp. BolN EW2_A01	nodule	MN943569	1	1	0	1	0	1	0	0	0	1	1	1	1	1
64	*Sphingomonas* sp. BceL EW2_D12	nodule	MN943526	1	1	0	1	1	1	0	1	1	1	1	1	1	1
65	*Stenotrophomonas maltophilia* BolN EW2_D5	nodule	MN943565	1	1	0	1	1	1	0	1	1	1	1	1	1	1
66	*Stenotrophomonas maltophilia* BolN EW3_G01	nodule	MN943566	1	1	0	1	1	0	1	1	1	1	1	1	1	1
67	*Stenotrophomonas* sp. BolN EW3_B03	nodule	MN943567	1	1	1	1	1	1	1	1	1	1	1	1	1	1
68	*Bacillus cereus* SatN EW4	nodule	MN943606	1	1	0	1	1	1	1	1	1	1	1	1	0	0
69	*Stenotrophomonas maltophilia* SatN EW3	nodule	MN943607	1	1	0	1	1	0	1	1	1	1	1	1	1	1
70	*Pseudomonas putida* SatN EW1	nodule	MN943608	1	1	1	1	1	1	1	1	1	1	1	1	1	1
71	*Pseudomonas putida* SatN EW2	nodule	MN943609	1	1	0	1	1	1	1	0	0	1	1	1	0	0
72	*Bacillus megaterium* BceL EW1_D03	leaf	MN943521	1	1	1	1	0	1	0	0	0	0	0	1	0	0
73	*Bacillus megaterium* BceL EW1_G02	leaf	MN943522	1	1	1	1	0	1	1	1	1	1	1	1	1	1
74	*Bacillus megaterium* BceL EW1_G04	leaf	MN943525	1	1	0	1	1	1	1	0	0	1	0	1	1	1
75	*Bacillus megaterium* BolL EW1_A02	leaf	MN943500	1	1	1	1	1	1	1	0	0	1	0	0	0	0
76	*Bacillus megaterium* BolL EW1_G05	leaf	MN943501	1	1	0	1	1	1	1	0	0	1	1	1	1	1

No	Bacterial strain ID	*T. repens* isol. source	NCBI accession number	Plant-growth promotion traits							Metal tolerance						
				IAA	sid	org acids	ACCD	acet	P solub	N-fix	Cd 0.4 mM	Cd 0.8 mM	Zn 0.6 mM	Zn 1.0 mM	Cu 0.4 mM	Cu 0.6 mM	Pb 2.0 mM
77	*Bacillus megaterium* BolL EW3_A02	leaf	MN943504	1	1	1	1	1	1	0	1	1	1	1	1	1	1
78	*Bacillus megaterium* BolL EW3_C03	leaf	MN943505	1	1	0	1	1	1	1	1	1	1	1	1	1	1
79	*Bacillus megaterium* BolL EW3_D03	leaf	MN943506	1	1	0	1	1	1	1	1	1	1	1	1	1	1
80	*Bacillus megaterium* BukL EW3_H07	leaf	MN943513	1	1	0	1	1	1	0	1	1	1	1	1	1	1
81	*Bacillus simplex* BukL EW2_F02	leaf	MN943518	0	1	0	1	0	0	1	1	1	1	1	1	1	1
82	*Bacillus subtilis* BceL EW2_A10	leaf	MN943524	1	1	0	1	1	1	0	0	0	0	0	0	0	0
83	*Bacillus* sp. BceL EW1_C01	leaf	MN943523	1	0	1	1	1	1	1	1	1	1	1	1	0	0
84	*Bacillus* sp. BolL EW1_E07	leaf	MN943502	1	1	0	1	1	1	1	1	1	1	1	1	1	1
85	*Bacillus* sp. BolL EW1_F06	leaf	MN943503	1	1	0	1	1	1	1	1	1	1	1	1	1	1
86	*Bacillus* sp. BukL EW3_B01	leaf	MN943514	1	1	1	1	0	1	1	1	1	1	1	1	1	1
87	*Bacillus* sp. BukL EW3_C01	leaf	MN943515	1	1	0	1	1	1	1	1	1	1	1	1	1	1
88	*Bacillus thuringiensis* BukL EW1_D12	leaf	MN943517	1	1	0	1	1	1	0	0	0	1	1	1	1	1
89	*Curtobacterium flaccumfaciens* BukL EW1_C12	leaf	MN943520	0	1	0	1	1	1	0	0	0	1	0	0	0	0
90	*Enterobacter* sp. BolL EW2_D03	leaf	MN943507	1	0	0	1	0	0	1	1	1	1	1	1	1	1
91	*Erwinia persicina* BceL EW3_H03	leaf	MN943540	1	1	0	0	1	1	1	1	1	1	1	1	1	1
92	*Erwinia* sp. BceL EW1_E06	leaf	MN943530	1	1	0	1	1	1	1	1	1	1	1	1	1	1
93	*Herbiconiux* sp. BceL EW1_D02	leaf	MN943529	1	1	0	1	0	1	0	0	0	1	0	1	0	0
94	*Methylobacterium* sp. BolL EW3_H01	leaf	MN943512	0	1	0	1	1	0	0	0	0	0	0	0	0	0
95	*Microbacterium* sp. BceL EW1_B07	leaf	MN943533	1	1	1	1	1	1	1	1	1	1	1	1	1	1
96	*Microbacterium* sp. BceL EW1_F07	leaf	MN943531	1	1	0	1	1	1	1	0	0	1	1	1	1	1
97	*Microbacterium* sp. BceL EW1_G06	leaf	MN943532	1	1	0	1	1	1	0	1	1	1	1	1	1	1
98	*Micrococcus* sp. BceL EW2_G04	leaf	MN943537	1	1	0	1	1	1	0	1	1	1	1	1	1	1
99	*Pantoea agglomerans* BceL EW1_A07	leaf	MN943534	1	1	1	1	1	1	0	0	0	1	0	1	1	1
100	*Pantoea agglomerans* BceL EW1_D07	leaf	MN943535	1	1	0	1	1	1	0	1	1	1	1	1	1	1
101	*Pantoea agglomerans* BceL EW1_D08	leaf	MN943536	1	1	0	1	1	1	0	0	0	0	0	0	0	0
102	*Pedobacter suwonensis* BceL EW2_C12	leaf	MN943539	1	1	0	1	1	1	0	0	0	0	0	0	0	0
103	Pseudomonas graminis BceL EW1_A01	leaf	MN943528	1	1	0	1	0	1	0	1	0	1	1	1	1	1

				Plant-growth promotion traits							Metal tolerance						
**No**	**Bacterial strain ID**	***T. repens* isol. source**	**NCBI accession number**	**IAA**	**sid**	**org acids**	**ACCD**	**acet**	**P solub**	**N-fix**	Cd 0.4 mM	Cd 0.8 mM	Zn 0.6 mM	Zn 1.0 mM	Cu 0.4 mM	Cu 0.6 mM	Pb 2.0 mM
104	*Pseudomonas graminis* BceL EW2_G05	leaf	MN943527	1	1	0	1	1	1	1	1	1	1	1	1	1	1
105	*Pseudomonas viridiflava* BukL EW2_B03	leaf	MN943519	1	1	1	0	1	1	1	1	1	0	0	0	0	0
106	*Sphingomonas phyllosphaerae* BukL EW2_E03	leaf	MN943516	1	0	0	1	1	1	1	1	1	1	1	1	1	1
107	*Sphingomonas* sp. BolL EW2_D07	leaf	MN943510	1	1	1	1	1	1	0	1	1	1	1	1	1	1
108	*Staphylococcus aureus* BolL EW1_C07	leaf	MN943511	1	1	1	1	1	1	0	1	1	1	1	1	1	1
109	*Stenotrophomonas maltophilia* BolL EW3_B05	leaf	MN943508	1	1	0	1	1	1	0	1	1	1	1	1	1	1
110	*Stenotrophomonas maltophilia* BolL EW3_C04	leaf	MN943509	1	1	0	1	0	1	0	1	1	1	1	1	1	1
111	*Bacillus megaterium* SatL EW2	leaf	MN943600	1	1	1	1	0	1	0	0	0	0	0	1	0	0
112	*Bacillus megaterium* SatL EW3	leaf	MN943601	1	1	1	1	0	1	1	1	1	1	1	1	1	1
113	*Stenotrophomonas maltophilia* SatL EW1	leaf	MN943602	1	1	0	1	1	1	0	1	1	1	1	1	1	1

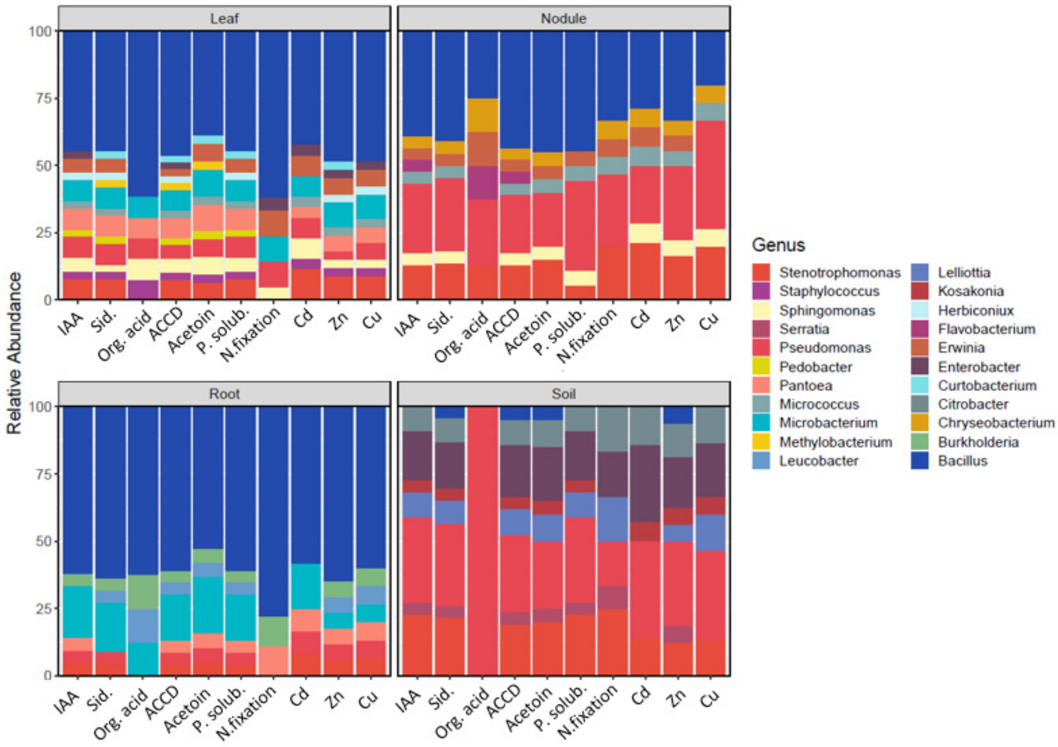

Figure 1. Relative abundance of the cultivable strains displaying *in vitro* PGP abilities or metal tolerance (0.4 mM Cd, 0.6 mM Zn, 0.4 mM Cu), distributed over the different compartments, leaf endosphere, nodule, root endosphere and rhizosphere soil of *T. repens*.

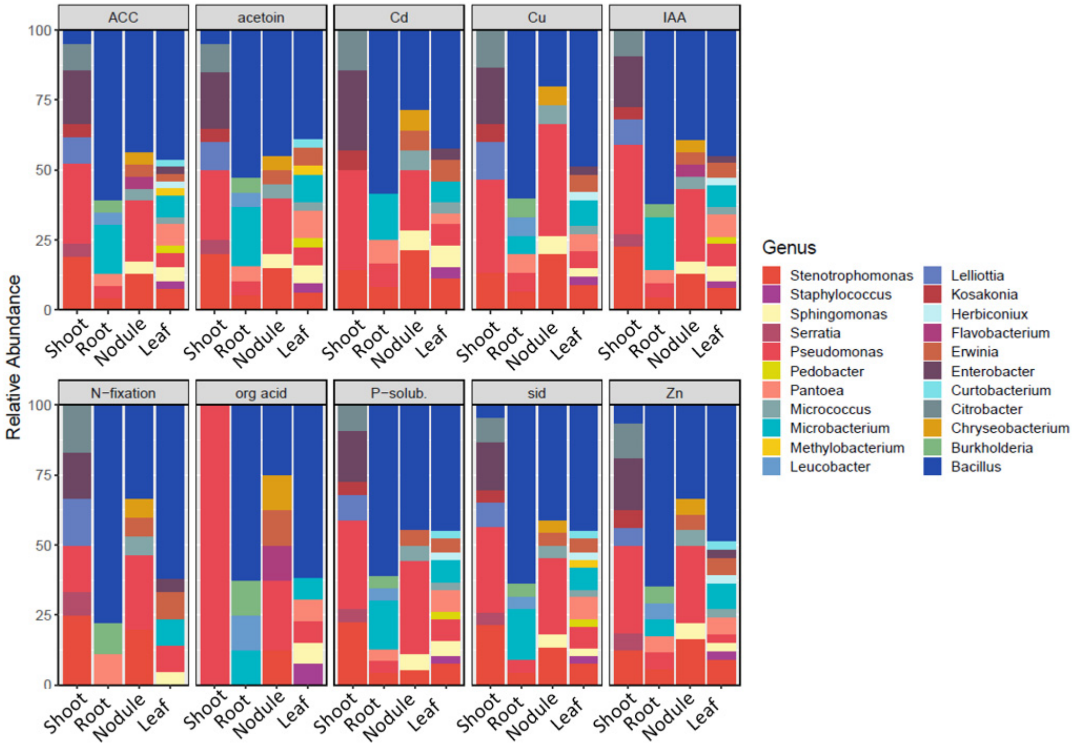

Figure 2. Relative abundance of the cultivable strains grouped per PGP trait and metal tolerance (0.4 mM Cd, 0.6 mM Zn, 0.4 mM Cu) and shown per compartment of *T. repens*.
